# Total Serum IgE in a Population-Based Study of Asian Children in Taiwan: Reference Value and Significance in the Diagnosis of Allergy

**DOI:** 10.1371/journal.pone.0080996

**Published:** 2013-11-20

**Authors:** Yu-Ling Tu, Su-Wei Chang, Hui-Ju Tsai, Li-Chen Chen, Wen-I Lee, Man-Chin Hua, Ju-Hui Cheng, Liang-Shiou Ou, Kuo-Wei Yeh, Jing-Long Huang, Tsung-Chieh Yao, for the PATCH study group

**Affiliations:** 1 Division of Allergy, Asthma, and Rheumatology, Department of Pediatrics, Chang Gung Memorial Hospital and Chang Gung University College of Medicine, Taoyuan, Taiwan; 2 Community Medicine Research Center, Chang Gung Memorial Hospital at Keelung, Keelung, Taiwan; 3 Graduate Institute of Clinical Medical Sciences, Chang Gung University College of Medicine, Taoyuan, Taiwan; 4 Clinical Informatics and Medical Statistics Research Center, Chang Gung University College of Medicine, Taoyuan, Taiwan; 5 Division of Biostatistics and Bioinformatics, Institutes of Population Health Sciences, National Health Research Institutes, Miaoli, Taiwan; 6 Department of Pediatrics, Feinberg School of Medicine, Northwestern University, Chicago, Illinois, United States of America; 7 Department of Pediatrics, Chang Gung Memorial Hospital at Keelung, Keelung, Taiwan; Cincinnati Children's Hospital Medical center, United States of America

## Abstract

**Background:**

Total serum immunoglobulin (IgE) test is usually performed to aid in the diagnosis of allergic diseases, but its reference values may vary among people of different ethnic backgrounds.

**Objectives:**

To establish reference values of total IgE in Asian children and to assess their significance in the diagnosis of atopy and allergic diseases.

**Study design:**

1321 Asian children aged 5-18 years in the Prediction of Allergies in Taiwanese CHildren (PATCH) study, a population-based cohort, were evaluated for total and specific IgE by ImmunoCAP and Phadiatop Infant, respectively.

**Results:**

Male, atopy, allergic diseases, recent symptoms of upper respiratory infection, and lower FEV_1_/FVC, were associated with higher total IgE levels in univariate analyses. Multivariate analysis revealed that atopy was the single most important determinant explaining 66.1% of the variability of total IgE levels in this population. The area under the receiver-operator characteristic (ROC) curve of total IgE for diagnosing atopy, asthma, rhinitis, and eczema were 0.92, 0.72, 0.70, and 0.70, respectively. The sensitivity, specificity, and positive and negative predictive values of total IgE at the optimal cutoff of 77.7 kU/L on the ROC curve for diagnosing atopy were 82.3%, 87.1%, 89.5%, and 78.6%, respectively. The corresponding values using the upper 95% CI of total IgE (164.3 kU/L) in non-atopic children were 61.2%, 95.0%, 94.3%, and 64.6%, respectively; whereas a customary cutoff (100 kU/L) provided accuracy between that of the aforementioned two cutoffs. Total IgE at the cutoff of 77.7 kU/L provided modest sensitivity and specificity (49.0%-78.3%) for diagnosing allergic diseases, but had high negative predictive values (84.2%-97.9%).

**Conclusions:**

Total serum IgE discriminates Asian children with and without atopy independent of allergic symptoms, with an optimal cutoff of 77.7 kU/L. The study confirms the insufficient diagnostic accuracy of total IgE alone to detect allergic diseases, but low total IgE levels may help exclude allergic diseases.

## Introduction

Serum immunoglobulin E (IgE) is the immunoglobulin mediating allergic sensitization to various allergens. Several studies have shown a quantitative relationship between serum IgE and atopy or various allergic diseases [1]. IgE levels have been an important part in assessing patients with established or suspected allergic disease for many years [2,3], although inadequate sensitivity has been reported [4]. Since there has been an increase in prevalence of allergic diseases all over the world [5,6,7], and both physicians and parents are eager to obtain prompt identification of children with allergic diseases, the applications of serum total IgE and its correlations with atopy and allergic diseases remains a focus of research interests [4,8,9].

Previous studies have demonstrated ethnic differences in total IgE levels [2,10]. Thus the reliability of total IgE as a diagnostic criterion of allergic diseases depends on the establishment of valid reference values for particular populations. Although several studies have been conducted on Western children to establish the reference values of serum total IgE levels and determine its application as a diagnostic test for allergic disease [2,3,4,11,12,13,14], few data are available in Asian children.

This study aimed to establish reference values of total IgE in a large population-based sample of Asian children, and to assess the significance of these values in the diagnosis of atopy (allergic sensitization) and allergic diseases.

## Methods

### Study subjects

**Figure 1:**
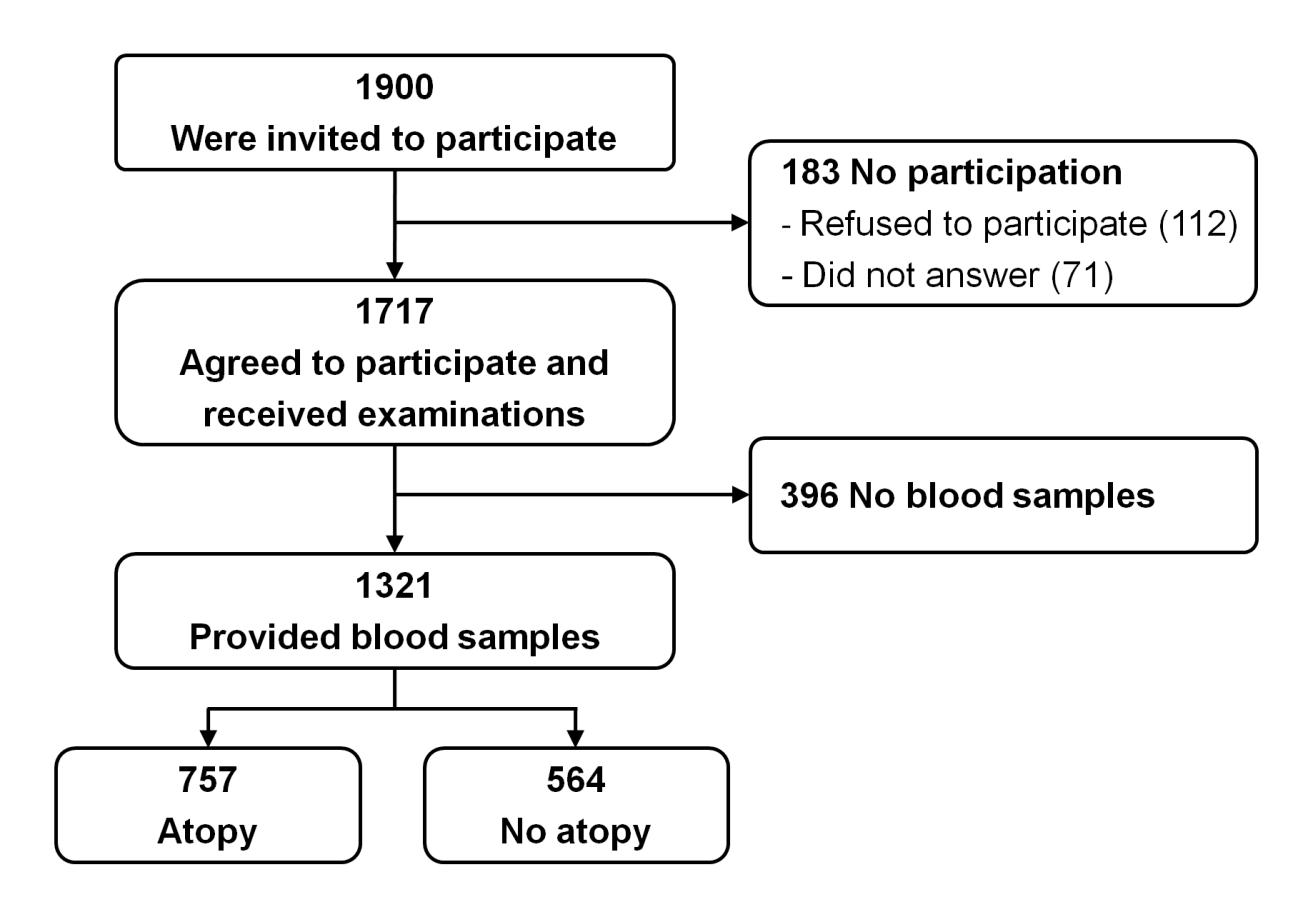
Schematic presentation of the recruitment process of the study subjects.

The study was a part of the Prediction of Allergies in Taiwanese CHildren (PATCH) study, a population-based prospective cohort study that was launched in 2007 to investigate the epidemiology and predictive factors of allergies and asthma in children [7,15,16]. Briefly, study participants in the current study were recruited from a school-based sample of 5351 children (2616 boys, 48.9%; age, 10.4±2.9 years) in an International Study of Asthma and Allergies in Childhood (ISAAC) epidemiologic survey. A random sample of 1900 children were invited to participate, and 1717 (90.4%) agreed. Parents of these 1,717 children answered questionnaires regarding demographic data, general health information, and questions on clinical symptoms and diagnosis of allergic diseases. Fraction of exhaled nitric oxide (FeNO) measurements and pulmonary function tests were performed, and if parents agreed to blood sampling, blood was collected for total and allergen-specific serum IgE. Serum total and specific IgE levels were measured successfully in 1,321 (76.9%) of 1717 subjects. There was no significant difference in terms of age, sex, and prevalence of allergic diseases between these 1321 subjects who received examinations and provided blood samples and the original 5351 cohort members, indicating a sampling cohort representative of the general population. The subject flow diagram is presented in Figure 1.

Weights and heights were measured according to standard protocols. Body mass index (BMI) was defined as weight (kg) divided by the height squared (m^2^) whereas body surface area (BSA) was defined as the square root of product of weight (kg) times height (cm) divided by 3600. This study was approved by the Institutional Review Board of Chang Gung Memorial Hospital and the parents of each subject provided written informed consent.

### Total and allergen-specific serum immunoglobulin E

The serum level of total IgE was determined by ImmunoCAP^®^ (Phadia, Uppsala, Sweden). Specific IgE was determined by a commercial assay for IgE (ImmunoCAP^®^ Phadiatop Infant; Phadia) against the most common inhalant and food allergens (i.e. house dust mite, cat, dog, birch, timothy, ragweed, wall pellitory, egg white, cow’s milk, peanut and shrimp) [17]. The Phadiatop Infant results were expressed on a scale of classes 0–6, with the following cutoff values: 0, 0.35, 0.70, 3.5, 17.5, 50, and 100 PAU/L.

### Exhaled nitric oxide and pulmonary function

All subjects with available serum total and specific IgE received pulmonary function tests by spirometry (Spirolab II**^®^**, Medical International Research, Roma, Italy) and online FeNO measurements by chemiluminescence analyzer (CLD 88sp NO analyzer^®^, Ecomedics, Duernten, Switzerland) according to the American Thoracic Sociey/European Respiratory Society recommendations [18,19].

### Definitions of phenotypes

 Allergic symptoms and diagnosis of allergic disease were assessed using a modified International Study of Asthma and Allergies in Childhood (ISAAC) questionnaire [20]. Asthma was defined as ever having asthma and either the occurrence of wheeze in the last 12 months or current use of asthma medications. Rhinitis and eczema were defined as ever having the two diseases, respectively, and either the presence of symptoms in the last 12 months or current use of medication for the two diseases, respectively. Atopy was defined as a positive Phadiatop Infant test result (≧0.35 PAU/L; class 1–6).

### Statistical analysis

The total IgE levels appeared to be log-normally distributed and were therefore logarithmically transformed for analysis. The results were presented as back-transformed values [i.e. geometric means and 95% confidence intervals (CI)]. Univariate analyses were performed using simple linear regression and unpaired t-tests to assess associations between log-transformed total IgE and the following variables: age, anthropometric measurements, pulmonary function variables, sex, allergic sensitization, symptoms of upper respiratory infection (URI) in the past two weeks, passive smoking, preterm birth. Variables with a *P*-value < 0.1 in univariate analyses were included in the multiple linear regression model. To exclude variables of multicollinearity, pairwise correlations between the potential explanatory variables were examined. Collinearity diagnostic statistics, including the tolerance and variance inflation factors (VIF), for each variable were also calculated to measure the impact of multicollinearity. Receiver-operator characteristic (ROC) curves were generated to assess the overall validity of total IgE for discriminating atopy, asthma, rhinitis, or eczema, respectively. All data analyses were performed using the SPSS statistical package version 15.0 for Windows (SPSS, Chicago, IL, USA).

## Results

**Figure 2:**
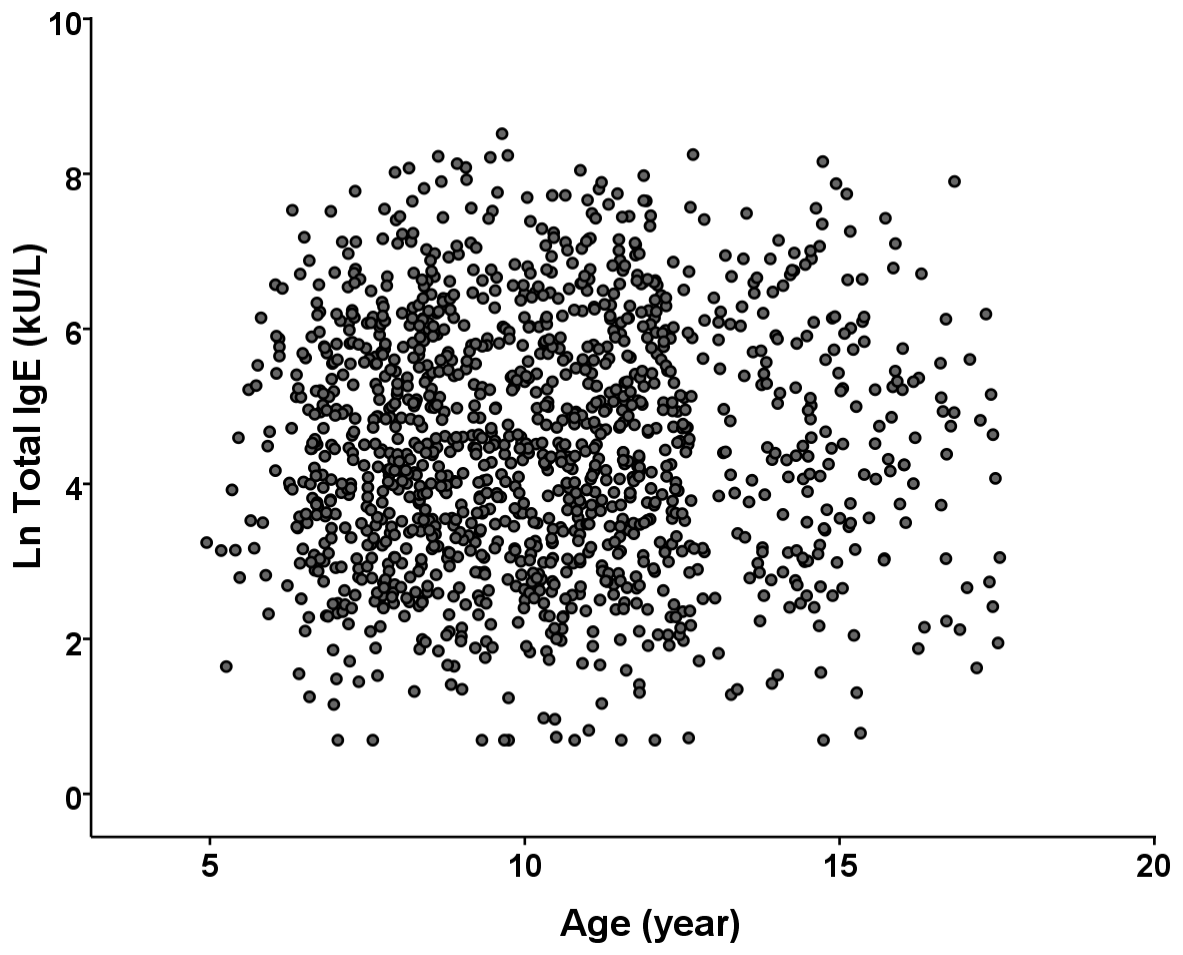
Distribution of total IgE levels in the study subjects by age.

The characteristics of the 1,321 subjects (644 boys; age: 10.3 ± 2.7 years [range 5-18]) are shown in Table 1 and Table 2. The numbers of subjects aged 5-7 years, 8-10 years, 11-13 years, and ≥14 years were 294, 526, 351, and 150, respectively. Figure 2 showed the distribution of total IgE levels in the study subjects by age. The geometric mean total IgE level in the study population was 91.1 kU/L (95% CI, 83.5-99.4). 757 atopic subjects (410 boys and 347 girls) and 564 non-atopic subjects (234 boys and 330 girls) were identified, respectively. The geometric mean total IgE level in the subgroup of 564 non-atopic subjects were 24.9 ppb (95% CI, 22.8-27.2).

**Table 1:** Characteristics of the study subjects and univariate analyses of factors (continuous variables) associated with total IgE.

**Variable**	**Mean ± SD**	**n**	**R**	***P*-value**
**Age (years)**	10.3 ± 2.7	1321	0.024	0.385
**Anthropometric measurement**				
Height (cm)	138.9 ± 14.8	1321	0.019	0.483
Weight (kg)	37.2 ± 13.5	1321	0.032	0.242
Body mass index (kg/m^2^)	18.7 ± 3.6	1321	0.036	0.194
Body surface area (m^2^)	1.19 ± 0.27	1321	0.029	0.291
**FeNO (ppb)**	25.9 ± 23.8	1278	0.551	**< 0.001**
**Pulmonary function**				
FVC (L)	2.09 ± 0.67	1290	0.030	0.285
FEV_1_ (L)	1.82 ± 0.58	1290	0.020	0.469
FEV_1_/FVC ratio (%)	87.3 ± 6.0	1290	-0.058	**0.038**
FEF_25-75_ (L/s)	2.23 ± 0.78	1290	-0.005	0.864
FVC % predicted (%)	91.3 ± 11.3	1290	-0.006	0.816
FEV_1_/FVC % predicted (%)	97.5 ± 6.8	1290	-0.041	0.145
FEF_25-75_ % predicted (%)	90.1 ± 19.2	1290	-0.029	0.302

IgE, immunoglobulin E; FeNO, fraction of exhaled nitric oxide; FVC, forced vital capacity; FEV_1_, forced expiratory volume in one second; FEF, forced expiratory flow.

**Table 2:** Characteristics of the study subjects and univariate analyses of factors (categorical variables) associated with total IgE.

			**IgE, Geometric mean (95% CI)**		
**Variable**	**%**	**N (yes/no)**	**Yes**	**No**	**P-value**
**Sex (male)**	48.8	644/677	112.8 (99.7-127.7)	74.4 (66.0-83.9)	**< 0.001**
Atopy*	57.3	757/564	239.5 (219.6-261.2)	27.5 (22.8-32.2)	**< 0.001**
**Asthma**	4.6	60/1239	282.8 (195.7-408.9)	84.5 (77.4-92.4)	**< 0.001**
**Rhinitis**	29.5	379/904	191.6 (165.1-222.3)	66.3 (59.9-73.4)	**< 0.001**
**Eczema**	6.8	87/1196	265.5 (191.3-368.5)	83.4 (76.2-91.2)	**< 0.001**
**Recent URI symptoms**	40.8	522/756	124.1 (108.0-142.6)	74.8 (66.9-83.6)	**< 0.001**
**Active smoking**	0.6	8/1279	197.0 (48.5-800.2)	91.7 (83.9-100.1)	0.179
**Passive smoking**	54.2	696/588	95.2 (84.7-107.1)	89.1 (78.1-101.7)	0.463
**Premature birth**	6.3	81/1196	89.3 (64.9-123.0)	92.5 (84.4-101.3)	0.851

* Atopy was defined as positive Phadiatop Infant test results (≥ 0.35 PAU/L).

IgE, immunoglobulin E; CI, confidence interval; URI, upper respiratory infection.

### Univariate and multivariate analyses of factors associated with serum total IgE

Univariate analyses were used to investigate the relationship of total IgE levels to the explanatory variables listed in Table 1 and Table 2. Total IgE levels significantly and positively correlated to FeNO level (*r* = 0.551, *P* < 0.001; Table 1). There was a significant but weak correlation between total IgE levels and FEV_1_/FVC ratio (*r* = -0.058, *P* =0.039; Table 1). There were significant differences in total IgE levels between subjects grouped by sex, allergic diseases (i.e. asthma, rhinitis, and eczema), allergic sensitization, and recent URI symptoms in the past two weeks (Table 2). There was no significant correlation between total IgE levels and age, height, weight, BMI, BSA, smoking, or premature birth.

 Boys had significant higher total IgE levels than the girls (geometric mean and 95% CI, 112.8 [99.7-127.7] vs. 74.4 [66.0-83.9]; *P* < 0.001; Table 2). Of note is that the prevalence of asthma, rhinitis, and eczema was significantly higher in boys (5.8%, 36.5%, and 8.6%, respectively) than in girls (3.5%, 22.9%, and 5.1%, respectively) (all *P* < 0.05). Asthma, rhinitis, and eczema were all associated with higher total IgE levels (all *P* < 0.001; Table 2). Atopic subjects had higher total IgE levels than non-atopic subjects (239.5 [219.6-261.2] vs. 27.5 [22.8.0-32.2]; *P* < 0.001; Table 2), with a positive correlation between total IgE levels and the height of Phadiatop infant titers (*r* = 0.669, *P* < 0.001). 

 Multivariate analysis of total IgE levels was performed using variables that had a *P*-value < 0.1 in univariate analyses. The highest ranked correlation was found between FeNO and rhinitis (Spearman's *r* = 0.332) and thus none of the candidate variables were excluded from the multivariate analysis. In the current study, the values of tolerance were all larger than 0.1 (ranging from 0.885 to 0.968) and the VIF values were all smaller than 2 (ranging from 1.033 to 1.130). Thus, multicollinearity was not considered a problem in this analysis. Only atopy was significantly and independently associated with total IgE levels in a multiple linear regression model which explained 66.1% of total variation in total IgE levels in this large population cohort of children (Table 3). The gradual increase of regression coefficients with increasing Phadiatop Infant classes suggested a dose response for the effect of atopy on the total IgE levels.

**Table 3:** Multivariate analyses of factors associated with total IgE.

**Variable**	**Coefficient (95% CI)**	***P*-value**
**Atopy**		
Phadiatop Infant class 6*	4.300 (3.962-4.637)	**< 0.001**
Phadiatop Infant class 5*	3.618 (3.403-3.834)	**< 0.001**
Phadiatop Infant class 4*	2.754 (2.575-2.933)	**< 0.001**
Phadiatop Infant class 3*	2.139 (1.974-2.304)	**< 0.001**
Phadiatop Infant class 2*	1.723 (1.559-1.887)	**< 0.001**
Phadiatop Infant class 1*	0.988 (0.803-1.174)	**< 0.001**
Phadiatop Infant class 0	-	-

R square = 0.661. Coefficient should be judged as the change of log total IgE when the variables change 1 unit.

* Compared with Phadiatop Infant class 0.

IgE, immunoglobulin E; CI: confidence interval.

### Determination of the reference values of total IgE

**Figure 3:**
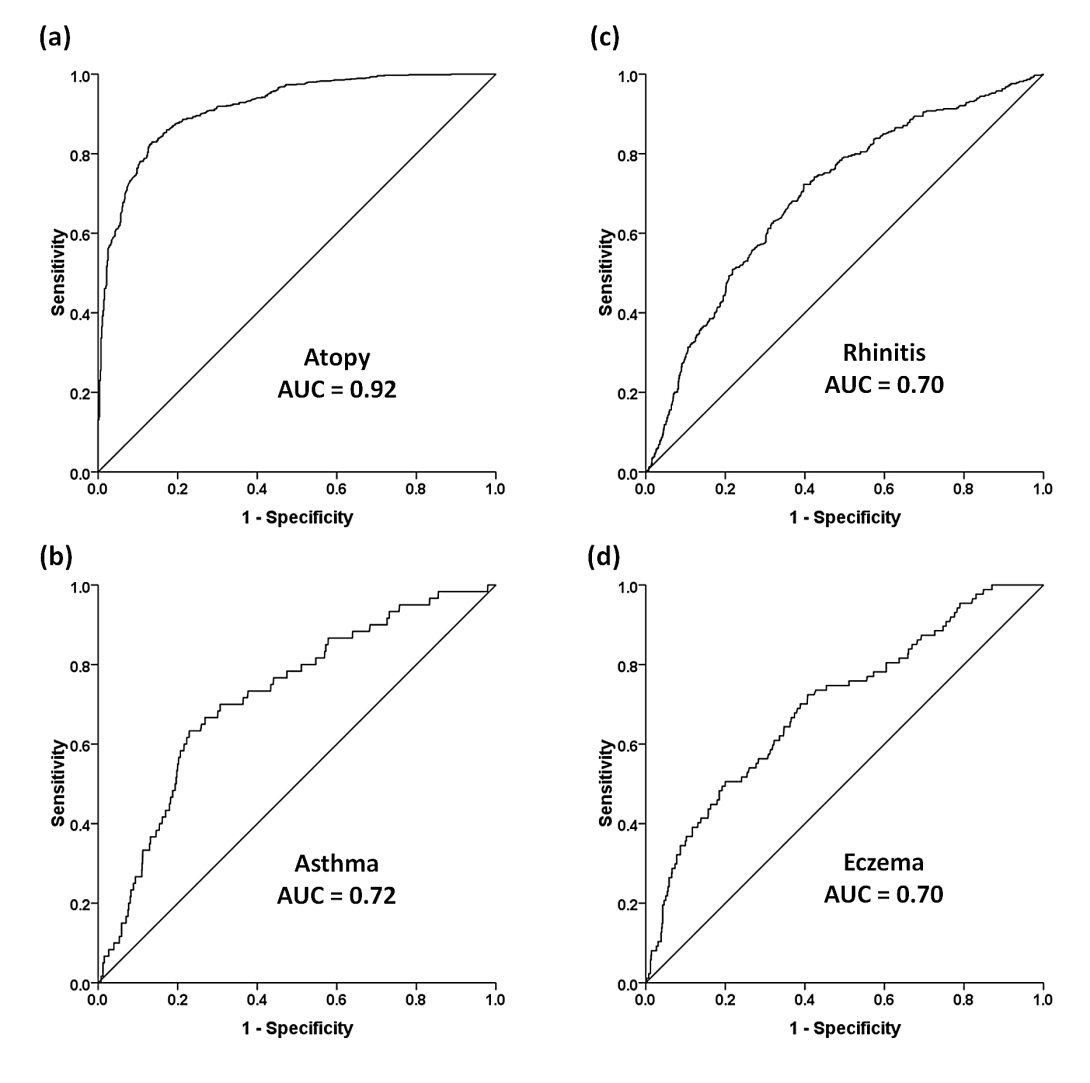
Receiver-operator characteristic (ROC) curves indicating the sensitivity and specificity of total IgE levels for predicting atopy (a), asthma (b), rhinitis (c), or eczema (d).

 A ROC curve was generated to determine the sensitivity and specificity of total IgE for discriminating subjects with and without atopy in this population (Figure 3a). The area under the ROC curve (AUC) (95% CI) was 0.92 (0.90-0.93). The highest combination of sensitivity and specificity was observed with a cutoff level of 77.7 kU/L (82.3% and 87.1%, respectively) for predicting atopy, with good positive and negative predictive values (PPV, 89.5% and NPV, 78.6%, respectively). The discriminative accuracy of total IgE was relatively modest for asthma, rhinitis and eczema (AUC [95% CI]: 0.72 [0.66-0.78], 0.70 [0.67-0.73], and 0.70 [0.64-0.76], respectively; Figure 3b, 3c, and 3d). The optimal cut-off levels on the ROC curves for diagnosing asthma, rhinitis, and eczema were 315.0 kU/L, 89.0 kU/L, and 122.0 kU/L, respectively.

### Validity of total IgE in discriminating atopy and allergic diseases

**Figure 4:**
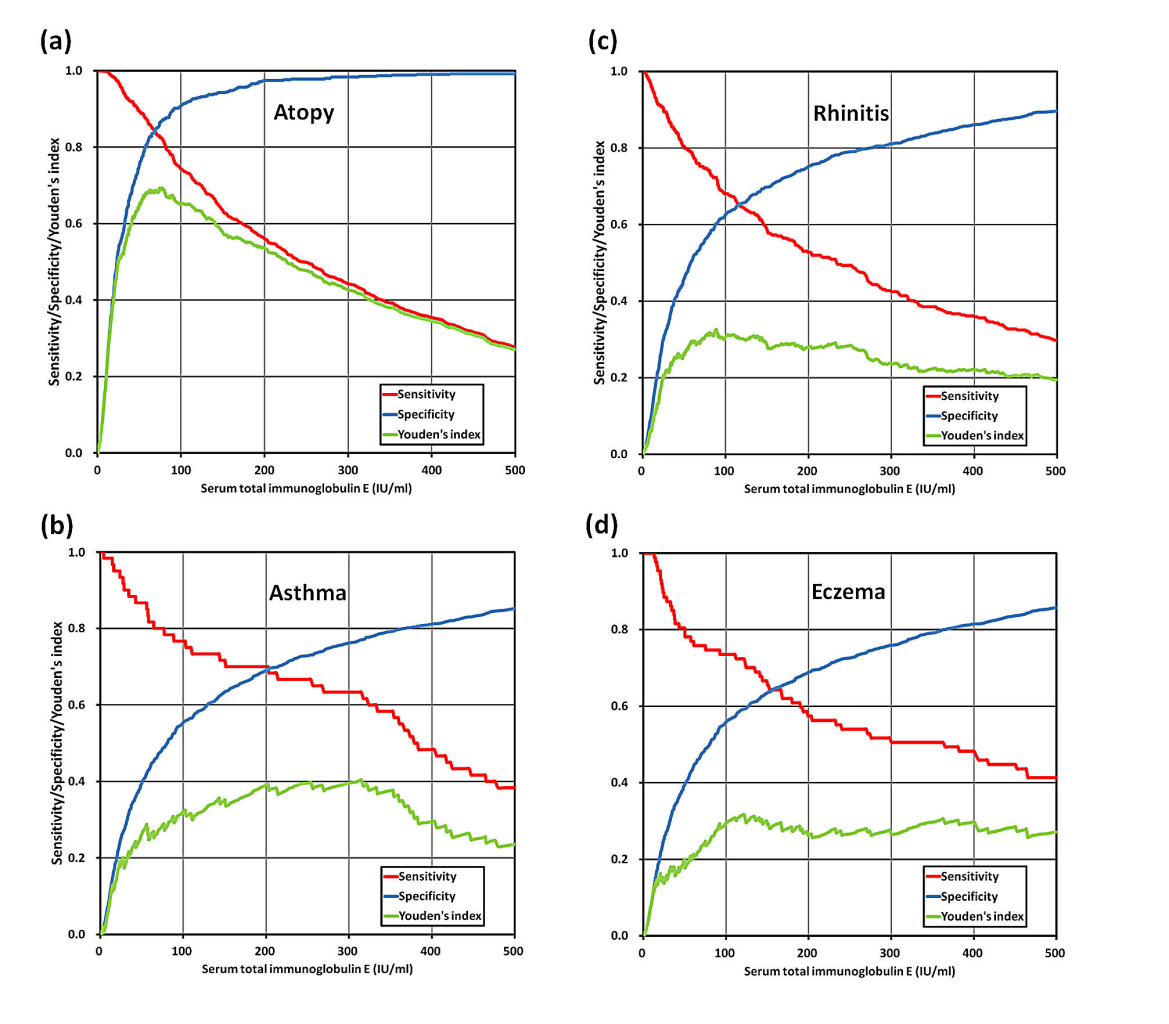
The sensitivity, specificity, and Youden's index of total IgE levels at different cutoff points as a diagnostic test for atopy (a), asthma (b), rhinitis (c), or eczema (d). The Youden's index, the sum of sensitivity and specificity minus one, is a commonly used measurement of overall diagnostic effectiveness.

Figure 4 showed the validity of serum total IgE levels at different cutoff points as a diagnostic test for atopy and allergic diseases. To investigate the capability of serum total IgE to discriminate children with and without atopy and allergic diseases in this population, we specifically compared three potential reference values: (1) 77.7 kU/L (the optimal cutoff on the ROC curve), (2) 164.3 kU/L (the upper 95% CI in 564 non-atopic subjects) and (3) 100 kU/L (a customary cutoff that is commonly used in clinical practice) [2,3]. The discriminative accuracy of serum total IgE at three cutoff values in the identification of atopy and three allergic diseases was shown in Table 4.

**Table 4:** Diagnostic sensitivity, specificity, positive and negative predictive values of total IgE at three reference values in the identification of atopy and allergic diseases.

**Phenotype**	**Reference value** * (**kU/L**)	**Sensitivity (%)**	**Specificity (%)**	**Positive predictive value (%)**	**Negative predictive value (%)**
Atopy	77.7	82.3	87.1	89.5	78.6
	164.3	61.2	95.0	94.3	64.6
	100	74.4	90.8	91.5	72.5
Asthma	77.7	78.3	49.0	6.9	97.9
	164.3	70.0	65.0	8.8	97.8
	100	76.7	55.4	7.7	98.0
Rhinitis	77.7	74.7	56.6	41.9	84.2
	164.3	57.0	71.3	45.5	79.8
	100	68.1	62.5	43.2	82.4
Eczema	77.7	74.7	49.2	9.7	96.4
	164.3	64.4	65.0	11.8	96.2
	100	73.6	55.8	10.8	96.7

* Three sets of reference values were compared: (1) 77.7 kU/L (the optimal cutoff on the ROC curve), (2) 164.3 kU/L (the upper 95% CI in non-atopic subjects) and (3) 100 kU/L (a customary cutoff that is commonly used in clinical practice).

Use of the 77.7 kU/L cutoff provided the best combination of diagnostic sensitivity and specificity; with such cutoff 82.3% of atopic children were detected. In contrast, the 164.3 kU/L and 100 kU/L cutoffs yielded relatively lower sensitivity of 61.2% and 74.4%, respectively, for atopy. The sensitivity and specificity of serum total IgE for diagnosing asthma, rhinitis and eczema was generally moderate but poor in some cases, ranging from 49.0% to 78.3%. Nonetheless serum total IgE level was associated with the very high negative predictive values (79.8%-98.0%) for these three allergic diseases.

We also performed sex stratified ROC analyses, which yielded very similar cutoff levels for boys and girls. Specifically, the optimal cutoff levels of total IgE on the ROC curves for diagnosing atopy were 79.7 kU/L and 77.7 kU/L for boys and girls, respectively. Table 5 showed the accuracy of sex-stratified optimal cutoffs on the ROC curves in the identification of atopy and three allergic diseases, which was nearly identical to that of the optimal cutoff of 77.7 kU/L on the ROC curve in the entire population.

**Table 5:** Diagnostic sensitivity, specificity, positive and negative predictive values of total IgE at sex-stratified optimal cutoffs on the ROC curves in the identification of atopy and allergic diseases.

**Group**	**Reference value** * (**kU/L**)	**Sensitivity (%)**	**Specificity(%)**	**Positive predictive value (%)**	**Negative predictive value (%)**
Atopy	79.7 (M)/77.7 (F)	82.2	87.4	89.8	78.5
Asthma	79.7 (M)/77.7 (F)	78.3	49.2	7.0	97.9
Rhinitis	79.7 (M)/77.7 (F)	74.4	56.9	41.1	84.1
Eczema	79.7 (M)/77.7 (F)	74.7	49.4	9.7	96.4

* The optimal cutoffs on the ROC curve for males and females were 79.7 and 77.7 kU/L, respectively.

M, male; F, female.

## Discussion

This large population-based study defines the reference value of total IgE levels in Asian children, which is independent of age and sex. Total IgE level discriminates Asian children with and without atopy independent of allergic symptoms, with an optimal cutoff of 77.7 kU/L. In contrast, this study confirms the insufficient diagnostic accuracy of total IgE levels alone to detect allergic diseases. The findings provide important information on interpreting total IgE levels in Asian children.

There has been considerable interest and debate regarding the validity of serum total IgE test as a diagnostic tool for atopy and allergic diseases. The current study assesses three sets of potential reference values of total IgE levels. The majority of reference values of total IgE levels published to date refer to the upper 95% CI of total IgE in non-atopic or non-allergic subjects [2,3,11,12,13]. However, the wide overlap of total IgE levels between atopic and nonatopic subjects leads to unsatisfactory performance of using the traditional upper 95% CI as cutoffs owing to their low diagnostic sensitivities, as demonstrated in the current and previous studies [2,11,12,13]. As shown in the present study, four in ten (38.8%) of atopic subjects have total IgE levels below the upper 95% CI of total IgE in non-atopic subjects (164.3 kU/L), and as such, will be misclassified as normal. Such findings argue against for the usefulness of upper 95% CI cutoffs of total IgE in clinical practice. In contrast, the commonly used customary cutoff of 100 kU/L yields a relatively higher, but not quite good enough, sensitivity of 74.4%. The PATCH child cohort, a population-based cohort of more than 1300 subjects with well-documented clinical and laboratory data and blood samples, allows us to employ the ROC curves to determine the optimal cutoff values as well as the sensitivities and specificities. Use of the optimal cutoff of 77.7 kU/L on the ROC curve provides the best combination of diagnostic sensitivity and specificity for atopy; with such cutoff 82.3% of atopic children are detected.

This study demonstrates the insufficient diagnostic accuracy of total IgE levels to detect allergic diseases regardless of which cutoff value is being used, indicating that total IgE is linked more to atopy than directly to symptoms. These results are in accordance with previous studies [2,4,11,12,13]. The current study demonstrates that the sensitivity and specificity of serum total IgE for diagnosing asthma, rhinitis and eczema was generally moderate but poor in some cases, ranging from 49.0% to 78.3%. Nonetheless, what is interesting is the very high NPVs (84.2%-97.9%) of total IgE levels at the cutoff of 77.7 kU/L for these three allergic diseases, implying that physicians could use low levels of total IgE in children with vague symptoms to exclude the diagnosis of allergic diseases.

This population-based study identifies that atopy is the single most important determinant of total IgE levels in Asian children, which explains 66.1% of total variation in total IgE levels in this population. Moreover, the clear dose-response correlation between total IgE levels and allergic sensitization in terms of Phadiatop Infant titers suggests that the relationship between IgE levels and atopy is not only qualitative but also quantitative. The reference value of total IgE levels established in this study is independent of age, sex and smoking. These results ensure that a simple set of reference value can be applied to children in the same age range. A number of previous studies, mostly conducted in adults, suggest the role of age, sex, or smoking on affecting total IgE levels, while other studies disagree [2,3,4,11,21]. The lack of association of age with total IgE in this study may be at least partially explained by the limited age range that we studied (5-18 years). In this study, sex is not independently associated with total IgE levels in the multivariate analyses, after taking atopy and clinical status into account. It is therefore very likely that the significant difference of total IgE levels between boys and girls in univariate analysis may in fact result from the higher prevalence of atopy and allergic diseases in boys. Given that sex-specific cutoff levels of total IgE adds little predictive accuracy in the identification of atopy and allergic diseases, one single cutoff value for children aged 5-18 years, regardless of sex, is therefore good enough for practical use. Although smoking has been reported to affect total IgE levels in adults, it is not possible to assess the influence of smoking on total IgE levels due to the very small percentage (0.6%) of active smokers in children in the current study.

The representative sampling of children in the community, large sample size, incorporation of objective markers of atopy all add strength to the results of this study. In addition, the population-based design in this study allows us to determine an optimal cutoff on the ROC curve, bringing the advantage that the ROC curve is independent of disease prevalence [22]. Apart from atopy, some conditions are well known to affect the values of total IgE, including parasite infections, immunodeficiencies, autoimmune diseases, and some neoplasms [23,24,25,26]. In our opinion, in a general population sample like ours, living in an area with exceptionally low prevalence of the above diseases, it is very unlikely that the results are significantly biased by the above factors.

In conclusion, this large population-based study provides important practical information regarding the interpretation of total IgE levels in Asian children. Total serum IgE test discriminates Asian children with and without atopy independent of allergic symptoms, with an optimal cutoff of 77.7 kU/L. The study confirms the insufficient diagnostic accuracy of total IgE levels alone to detect allergic diseases. Another important message for physicians is that low serum total IgE levels in children may help exclude the diagnosis of allergic diseases.
